# Combined double tarsal wedge osteotomy and transcuneiform osteotomy for correction of resistant clubfoot deformity (the “bean-shaped” foot)

**DOI:** 10.1007/s11832-014-0613-0

**Published:** 2014-10-04

**Authors:** Adham Elgeidi, Mazen Abulsaad

**Affiliations:** Mansoura School of Medicine, Mansoura University, Elgomhoria Street, P.O. Box 95, Mansoura, 35516 Egypt

**Keywords:** Clubfoot, Cuboid closing wedge, Medial cuneiform opening wedge, Transcuneiform osteotomy

## Abstract

**Purpose:**

The “bean-shaped foot” exhibits forefoot adduction and midfoot supination, which interfere with function because of poor foot placement. The purpose of the study is a retrospective evaluation of patients who underwent a combined double tarsal wedge osteotomy and transcuneiform osteotomy to correct such a deformity.

**Methods:**

Twenty-seven children with 35 idiopathic clubfeet were treated surgically by combined double tarsal wedge osteotomy (closing wedge cuboid osteotomy and opening wedge medial cuneiform osteotomy) and transcuneiform osteotomy between 2008 and 2012. The age of children at surgery ranged from 4 to 9 years. There were 19 boys and 8 girls. Pre- and postoperative X-rays were used, considering: on the AP radiograph, the calcaneo-fifth metatarsal angle and the talo-first metatarsal angle (indicators of forefoot adduction); on the lateral radiograph, the talo-first metatarsal angle (an indication of supination deformity) and calcaneo-first metatarsal angles (an indication of cavus deformity). These radiological parameters were compared with the clinical results.

**Results:**

Follow-up was conducted for 24–79 months following surgery. Clinical and radiographic improvements in forefoot position were achieved in all cases. An average improvement in the anteroposterior talo-first metatarsal angle of 21°, calcaneo-fifth metatarsal angle of 14°, lateral talo-first metatarsal angle of 10°, and lateral calcaneo-first metatarsal of 12° confirmed the clinically satisfactory correction in all feet. One patient had a wound infection postoperatively, which resolved with removal of the wires and administration of oral antibiotics. Eight patients followed up for more than 5 years had no deterioration of results.

**Conclusions:**

Combined double tarsal wedge osteotomy as well as transcuneiform osteotomy is an effective and safe procedure for lasting correction of the bean-shaped foot.

## Introduction

**Fig. 1:**
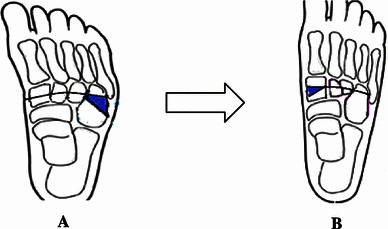
A drawing of the foot shows correction of the bean-shaped foot after transcuneiform osteotomy and removal of a wedge from the cuboid (**a**), inserting it into the medial cuneiform (**b**)

The "bean-shaped foot" is a term used to describe a clinical deformity of forefoot adduction and midfoot supination. It often causes functional disability due to poor foot placement. There is no consensus on the management of such deformities persisting as a result of a failed treatment of an idiopathic clubfoot in patients who have not reached skeletal maturity [1]. The crucial principle involved in understanding the physiopathology of forefoot adduction states that there is an imbalance between an elongated lateral column and shortened medial column [2]. Although Ponseti serial casting techniques, soft tissue releases, and tendon transfers have met with success in many cases [3], a group of resistant clubfoot patients remains that requires a bony procedure to correct the residual deformity. We believe that treating this condition should involve a combination of lateral column shortening and medial column lengthening (to correct the forefoot adduction) associated with transcuneiform osteotomy (to correct the midfoot supination deformity) [1, 3, 4] (Fig. 1).

We present a retrospective study of patients who underwent a combined double tarsal wedge osteotomy (closing wedge cuboidal osteotomy and a medial cuneiform opening wedge osteotomy) and transcuneiform osteotomy to correct this deformity.

## Materials and methods

We retrospectively reviewed 27 children (35 feet) with resistant “bean-shaped” deformity after clubfoot surgery who were treated surgically by combined double tarsal wedge osteotomy (closing wedge cuboid osteotomy and opening wedge medial cuneiform osteotomy) and transcuneiform osteotomy between 2008 and 2012. Soft tissue releases were used when necessary (i.e., abductor hallucis and plantar fascia). Our inclusion criteria were idiopathic clubfeet patients with a previous clubfoot surgery that had failed to correct a severe deformity and ended up with a fixed (stiff) “bean-shaped” foot deformity. These deformities were resistant to Ponseti serial casting. Our exclusion criteria included patients with complex (atypical) clubfeet, patients who had additional significant procedures concomitant with osteotomies, patients who had undergone previous multiple surgical procedures, and patients who had any other anomaly, such as spina bifida or arthrogryposis.

Twenty-six of the feet had the primary posteromedial-lateral release through the Carroll two-incision approach, and nine had the posteromedial release through a “hockey-stick” medial incision. Age of children at surgery ranged from 4.3 to 9.2 years (mean 5.4). There were 19 boys and 8 girls. The resistant deformity was bilateral in 8 patients and unilateral in 19.

Preoperatively, all feet had significant forefoot adduction and supination without equinus deformity and most had mild hindfoot varus. The varus deformity was tested using the Coleman block test for assessment of flexibility. Most parents reported gait abnormalities, calluses, difficulty in fitting shoes, and abnormal shoe wear. Activity-related foot pain was noted in five patients.

We used the standardized radiological method and technique of Simons [5] to minimize errors in measurements. Pre- and postoperative weight-bearing X-rays were used, considering: on the AP radiograph, the talo-first metatarsal angle and the calcaneo-fifth metatarsal angle (indicators of forefoot adduction) [6, 7]; on the lateral radiograph, the talo-first metatarsal angle (an indication of supination deformity) and calcaneo-first metatarsal angles (an indication of cavus deformity) [7]. These radiological parameters were compared with the clinical results.

Postoperative evaluation was based on the modified criteria of Heyman et al. [8]. A satisfactory outcome was considered to be normal alignment of the forefoot or mild adduction, as assessed by the Bleck method [9], with parents and doctors pleased with the outcome, and an unsatisfactory outcome was considered to be moderate to severe deformity requiring further treatment, with parents and doctors not pleased with the outcome.

### Surgical procedure

**Fig. 2:**
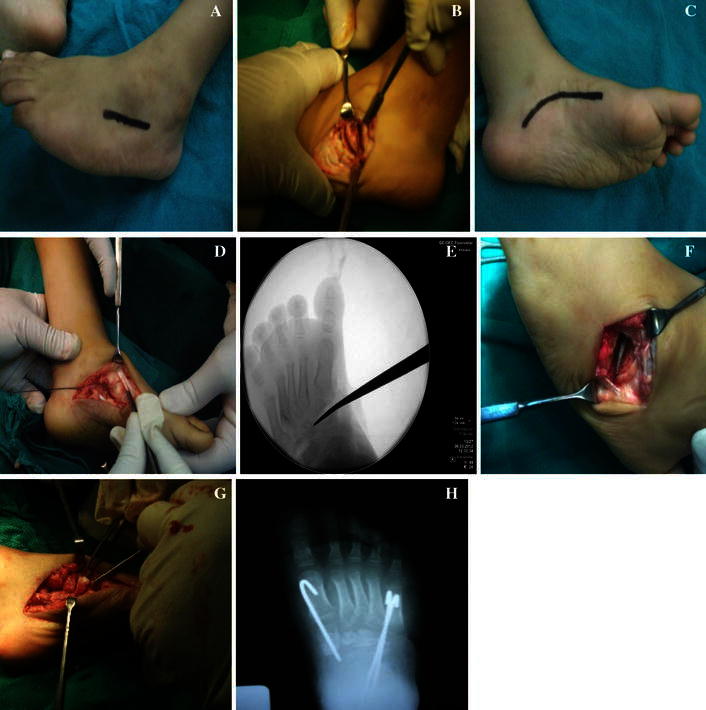
Intraoperative photographs: **a** lateral skin incision. **b** Appropriate dorsolaterally based wedge of the cuboid bone was removed. **c** Medial skin incision. **d** The tibialis anterior tendon was identified crossing over the medial cuneiform. **e** With the aid of an image intensifier, an osteotome was used to make a slightly curved osteotomy starting from the medial cuneiform. **f** The osteotome exiting at the apex of the cuboid osteotomy. **g** The wedge of bone taken from the cuboid bone was inserted into the medial cuneiform. **h** Fixation with K.W. wires

Under general anesthesia and sterile conditions, the patient was positioned supine on the surgical table. An above-knee tourniquet was used. A longitudinal lateral incision allowed reflection of the extensor digitorum brevis and exposure of the cuboid (Fig. 2a). The peroneal tendons were identified and retracted plantar. Using a micro-oscillating saw, a closing dorsolaterally based wedge of the cuboid bone was removed (Fig. 2b). Bone nibblers were used to ensure that the apex of the wedge extended to the lateral cuneiform. The skin incision was made on the medial aspect of the foot in accordance with the shape of its longitudinal arch (Fig. 2c). Previous surgical incisions were used whenever possible. A plantar fascial release and division of the abductor hallucis muscle were performed when necessary. Next, the tibialis anterior tendon was identified crossing over the medial cuneiform (Fig. 2d), and part of it was reflected dorsally to identify the cuneometatarsal first joints (without opening the joint capsule).

With the aid of an image intensifier, a single osteotomy cut of the medial cuneiform was performed using a micro-oscillating saw. Retractors were used to protect the soft tissues above and below the bones. An osteotome was used to make the transcuneiform osteotomy, with an osteotome exiting at the apex of the cuboid osteotomy (Fig. 2e, f). Rotation of the forefoot at this midfoot slighty curved osteotomy allows correction of the supination. The lateral cuboid wedge was closed and held with one or two wires. These usually traversed the base of the fifth metatarsal and transfixed the calcaneocuboid joint. A wedge of suitable size taken from the cuboid was inserted gently into the osteotomy of the medial cuneiform. The graft was secured with one or two wires passed through the base of the first metatarsal, distal fragment, graft, and proximal fragment (Fig. 2g, h). This combined with the closure of the lateral cuboid wedge corrected the adduction.

The wounds were dressed and closed without a drain. The wires were left outside the skin. The foot was immobilized in a below-the-knee cast, and weight-bearing was not permitted. At 2 weeks, the cast was changed after wound inspection. Four weeks later, the cast was changed and the wires removed. Total cast time was 12 weeks.

### Statistical analysis

Data were analyzed using SPSS (Statistical Package for Social Science) version 16. *P* **<** 0.05 was considered to be statistically significant.

## Results

Follow-up was conducted for 24–79 months following surgery. Clinically, all patients had satisfactory results. Parents and patients were pleased regarding better shoe fitting and the appearance of straight feet.

The radiographic improvement in different radiological angles confirmed the clinically satisfactory correction in all feet. The average improvement of adduction as measured by the anteroposterior talo-first metatarsal angle and calcaneo-fifth metatarsal angle was 21° and 14°. These changes were statistically significant comparing the pre- and postoperative values (*P* < 0.05).

**Fig. 3:**
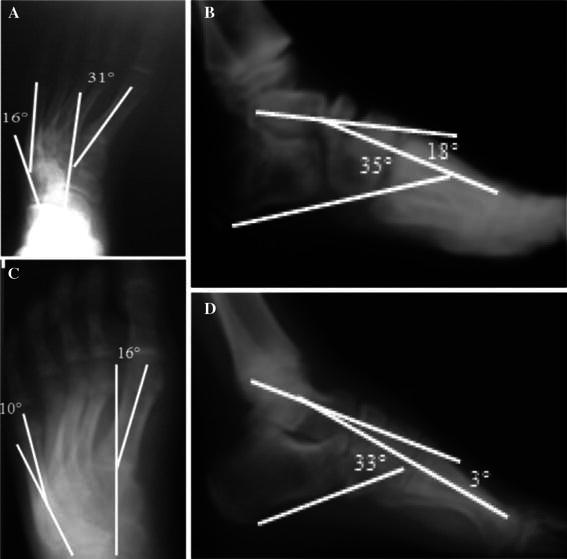
The preoperative anteroposterior (**a**) and lateral (**b**) radiographic view of the patient’s left foot is shown. The postoperative anteroposterior (**c**) and lateral (**d**) radiograph of the left foot is shown following this procedure, demonstrating correction of adduction in both views

The average improvement of supination and cavus as measured by the lateral talo-first metatarsal angle and lateral calcaneo-first metatarsal was 10° and 12°; however, these changes were not statistically significant (Table 1; Fig. 3).

**Table 1:** Radiographic parameters

Radiographic parameters	Preoperative		Postoperative		Average improvement (°)	*P* value
	Range (°)	Average (°)	Range (°)	Average (°)		
TMT-AP	18–42	24	16 to −8	3	21	<0.05
C-5th MT-AP	12–58	23	10 to −4	9	14	<0.05
TMT-LAT	14–33	21	4 to 17	11	10	<0.1
C-1st MT-LAT	29–64	51	8 to 49	39	12	<0.2

*TMT-AP* talo-first metatarsal angle in anteroposterior projection, *C-5th MT-AP* calcaneo-fifth metatarsal angle in anteroposterior projection, *TMT-LAT* talo-first metatarsal angle in lateral projection, *CMT* calcaneo-first metatarsal angle in lateral projection

One patient had wound infection postoperatively, which resolved with removal of wires and administration of oral antibiotics. Collapse of the graft did not occur in any patient. Eight patients followed up for more than 5 years had no deterioration in results.

## Discussion

The essential deformity in residual clubfoot is the disproportionate length between the medial and lateral columns of the foot [2]. Although the Ponseti serial casting technique has been effective in correcting even neglected clubfeet [10], surgical procedures may be needed to correct more rigid and structural deformities. In our study, we treated the “bean-shaped” deformities surgically by combined double tarsal wedge osteotomy and transcuneiform osteotomy after failure of Ponseti serial casting.

Numerous types of osteotomies and their combinations have been previously described for correction of forefoot adduction and midfoot supination. The Evans procedure of reestablishing the balance between the two columns by calcaneocuboid fusion may cause a reverse deformity with an abducted foot. It also did not correct adduction occurring distal to the talonavicular and calcaneocuboid joints or supination. In 1973, Lichtblau [11] suggested a resection of the anterior end of the calcaneus as a means of avoiding this complication. However, this acts on only one of the sides of the deformity, as happens with procedures that lengthen the medial column, such as the one described by Hoffman et al. [12] in 1984 and used by others [13, 14]. Medial column lengthening also has an additional problem because it requires harvesting a bone graft from another site. It also does not easily address the supination deformity. Napiontek et al. [15] in their series on opening wedge osteotomy of the medial cuneiform in the treatment of forefoot adduction reported 14 % overcorrection (forefoot abduction), and in one-quarter of the operated feet, the ceramic porous graft had to be removed.

The double osteotomy addresses the problem by lengthening the medial column using the wedge resected from the lateral column. It affects both sides of the deformity, permitting a better correction than an isolated procedure in just one column. In 1991, McHale and Lenhart [16] first described the combination of a shortening osteotomy of the cuboid and elongation of the cuneiform without transcuneiform osteotomy. Supported by cadaveric study, they suggested the cuboid closing wedge corrected the midfoot, whereas the cuboid and cuneiform osteotomies both contributed to the correction in the forefoot [16]. A semicircular tarsal osteotomy has been described by Gupta and Kumar [17]. This was not combined with closing cuboid or opening cuneiform osteotomies and thus does not seem to address the imbalance between the long lateral and short medial columns characteristic of these feet. In addition, their exposure through a transverse dorsal incision in feet with previous scarring would seem to involve risk.

Kose et al. [4], using cadaveric and clinical studies, reported a combination of three procedures, namely transmidtarsal, closing cuboidal, and opening cuneiform wedge osteotomy, on 10 feet with adduction and supination deformities and another three patients with cavovarus feet. The added advantage of the trans-midfoot procedure was to allow correction of the rotational component as it was centered at the apex of the deformity. Therefore, these combinations allowed corrections of three planes to be made simultaneously. However, this method did not address the residual hindfoot varus. Pohl and Nicol [1] adapted the technique by exiting the osteotome at the apex of the cuboid osteotomy and reported similar results with a reliable correction of adduction and supination.

Lourenco et al. [18] subsequently reported their series of treatment of residual adduction deformity in clubfeet by double osteotomy and advocated that surgery should be reserved for children older than 4 years of age when the medial cuneiform ossific nucleus is well developed. Using similar techniques, Gordon et al. [19] stated a success rate of 90 % improvement in both clinical and radiographic evaluation. They, too, recommended that the procedure should be reserved for patients aged 5 years or older.

In 2009, Mahadev et al. [3] described a corrective procedure for treatment of the "bean-shaped foot," combining a closing wedge cuboidal osteotomy and transmidfoot rotation procedure without a medial opening wedge osteotomy. They chose not to perform the medial opening wedge osteotomy since all of the children in their series were younger than 5 years old with a less than well-developed medial cuneiform ossific nucleus. They believed the medial cuneiform opening wedge osteotomy should still be performed in older children once the ossific nucleus has become well defined for a better correction of forefoot adduction deformities.

The amount of correction of the adduction obtained in the current study, measured by the anterior talo-first metatarsal (21º) angle and calcaneo-fifth metatarsal angle (14º), was comparable to others radiographically [1, 3, 19, 20], but better than that obtained by McHale and Lenhart [16], reported in 1991, whose average correction of the anterior talo-first metatarsal was 9º, probably because they had patients with inadequate correction of the hindfoot with a low talocalcaneal index.

The average improvement of supination and cavus obtained in our seies, as measured by the lateral talo-first metatarsal (10º) and the lateral calcaneo-first metatarsal angle (12º), was not statistically significant. This is in agreement with the results of Pohl and Nicol [1]. Therefore, it is difficult to document both supination and cavus deformities and their correction.

## Conclusion

In conclusion, combined double tarsal wedge osteotomy as well as transcuneiform osteotomy is an effective and safe procedure for lasting correction of the bean-shaped foot. We believe that this approach has produced excellent results, with a straight foot. Correction is easily achieved and complications are few. There was no deterioration of results in patients followed up for more than 5 years.
